# Agitation and aggression in people living with dementia and mild cognitive impairment in shared-housing arrangements – validation of a German version of the Cohen-Mansfield Agitation Inventory-Short Form (CMAI-SF)

**DOI:** 10.1186/s12955-023-02132-y

**Published:** 2023-05-29

**Authors:** André Kratzer, Jennifer Scheel-Barteit, Janissa Altona, Karin Wolf-Ostermann, Elmar Graessel, Carolin Donath

**Affiliations:** 1 Center for Health Services Research in Medicine, Department of Psychiatry and Psychotherapy, Uniklinikum Erlangen, Friedrich-Alexander-Universität Erlangen-Nürnberg (FAU), Schwabachanlage 6, D-91054 Erlangen, Germany; 2 Institute of General Practice, Uniklinikum Erlangen, Friedrich-Alexander-Universität Erlangen-Nürnberg (FAU), Universitätsstr. 29, D-91054 Erlangen, Germany; 3 Institute for Public Health and Nursing Science (IPP), University of Bremen, Grazer Str. 4, D-28359 Bremen, Germany; 4 Health Sciences Bremen, Bremen, Germany

**Keywords:** CMAI, CMAI-SF, Cohen-Mansfield Agitation Inventory, Validity, Reliability, Agitation, Aggression, Dementia, Mild cognitive impairment, Shared-housing arrangements

## Abstract

**Background:**

The Cohen-Mansfield Agitation Inventory-Short Form (CMAI-SF) is a 14-item scale for assessing agitation and aggression, derived from the original 29-item CMAI, and completed by a proxy. Because the CMAI-SF has not yet been validated in German language, the aim of this study is to explore its construct validity.

**Methods:**

Baseline data from a cluster-randomized trial to evaluate a non-pharmacological complex intervention for people living with dementia (PlwD) and mild cognitive impairment (MCI) were analyzed. The study sample consisted of 97 shared-housing arrangements (SHAs) in Germany, comprising *N* = 341 residents with mild to severe dementia and MCI. Trained nursing staff collected data by proxy-rating the CMAI-SF, Neuropsychiatric Inventory-Nursing Home Version (NPI-NH), and QUALIDEM. They also conducted the Mini-Mental State Examination (MMSE) and the Montreal Cognitive Assessment (MoCA).

**Results:**

In an exploratory factor analysis, three factors emerged: “aggressive behavior”, “verbally agitated behavior”, and “physically non-aggressive behavior”. The CMAI-SF total score showed good internal consistency (α = .85), and the factors themselves showed adequate internal consistency (α = .75/.76/.73). The CMAI-SF showed convergent validity with the NPI-NH agitation item (*r* = .66) and the NPI-NH “agitation & restless behavior” factor (*r* = .82). Discriminant validity was confirmed by a low (*r* = .28) correlation with the NPI-NH apathy item. Quality of life decreased significantly with agitation, as the CMAI-SF showed a moderate negative correlation with the QUALIDEM total score (*r* = -.35).

**Conclusions:**

The 14-item CMAI-SF is a time-efficient, reliable, and valid assessment instrument. Three factors emerged that were similar to those already found in nursing home samples for the original CMAI and the CMAI-SF and in day care samples for the CMAI-SF. The findings provide preliminary evidence that the CMAI-SF can be used instead of the CMAI to reduce time, costs, and burden in future trials.

**Trial registration:**

The DemWG study from which data were used to draft this manuscript was prospectively registered on 16 July 2019 at ISRCTN registry (ISRCTN89825211).

## Introduction

Nearly all people living with dementia (PlwD) will be affected by behavioral and psychological symptoms of dementia (BPSD) over the course of their disease [1]. BPSD include agitation and aggression but also other non-cognitive symptoms of dementia, such as depression, anxiety, delusions, or hallucinations. For PlwD, agitation in particular is a common complex of symptoms: prevalence rates range from 33 to 45% for PlwD in nursing homes, and the prevalence increases in more progredient illness states [2]. Occasionally, a higher prevalence (i.e. 85% for at least one agitation symptom) is noted [3]. Agitated and aggressive behaviors challenge both the PlwD and their caregivers [4, 5]. A recent meta-analysis showed that there are efficacious non-pharmacological interventions for agitation and aggression in PlwD [6]. However, BPSD still constitute a common cause of hospitalization for PlwD [7, 8], which in turn is associated with negative consequences, such as the risk of institutionalization and declines in mental, cognitive, and physical capabilities [9]. Furthermore, hospitalization can stabilize or worsen agitation and aggression, most likely by increasing unmet needs or distress [9]. In order to carry out valid and reliable research on agitation and aggression and its consequences, the sound scientific measurement of these constructs is a prerequisite.

Early efforts to describe and categorize different behaviors reflecting agitation and aggression were made by Cohen-Mansfield et al. [10], who described 29 behaviors in the Cohen-Mansfield Agitation Inventory (CMAI) each rated on a 7-point frequency scale [10, 11]. These behaviors were observed by professional caregivers in nursing homes and clustered into the three factors (I) “aggressive behaviors,” such as pushing, scratching, and grabbing, (II) “physically non-aggressive behaviors,” such as handlings things inappropriately, as well as (III) “verbally agitated behaviors,” such as complaining, negativism, and screaming [10]. Some later validation studies on older people with or without dementia in different countries were able to replicate the original three factor structure [12, 13], while some other studies with nursing home residents suggested the categorization of the 29 behaviors into four categories (an additional Factor IV called “hiding and hoarding” comprising the items “hiding” and “hoarding”) [14–16].

Beyond that, Cohen-Mansfield et al. [17] developed the community version of the CMAI (CMAI-C), which is an expanded version of the CMAI including 36 items specifically adapted to day-care population. Using the CMAI-C, Cohen-Mansfield et al. [17] found that in semi-inpatient structures, such as day-care centers, agitation behaviors rated by family caregivers clustered into the three original categories for nursing home residents found by Cohen-Mansfield et al. [10], whereas agitation behaviors rated by staff clustered into the three categories: “physically non-aggressive behaviors” (corresponding with the original Factor II), “verbally agitated behaviors” (corresponding with the original Factor III), and “verbal aggressive behaviors” (behaviors that originally fell under Factor I). Thus, the concept was adapted to semi-inpatient and community-settings, and four possible behavioral categories were named: “verbally non-aggressive behaviors (VNAB),” “verbally aggressive behaviors (VAGB),” “physically non-aggressive behaviors (PNAB),” and “physically aggressive behaviors (PAGB).” By contrast, Weiner et al. [18] could not satisfactorily replicate the suggested factor structure in community-dwelling PlwD using the CMAI-C. Their results suggested that agitation behaviors in community-dwelling PlwD cluster unstable into the “verbally aggressive behavior,” “verbally agitated behavior,” and “hiding and hoarding” categories, whereby many physically aggressive behaviors do not occur or rarely occur in community-dwelling PlwD. The latter was also reported by Koss et al. [19]. As a consequence, Weiner et al. [18] suggested that the CMAI-C should be used instead as an overall measure and that subscale scoring does not seem applicable in community-dwelling PlwD.

The retest reliability of the CMAI-C (*r* = 0.83) [19] and the CMAI (*r *= 0.85) [13] was reported to be acceptable in PlwD. Studies exploring construct validity including factor structure of the long form [10, 12–16] and the CMAI-C [17, 18] in different settings are displayed in Table 1.

**Table 1:** Factor structures of previous studies on different CMAI versions in different settings

Version	Authors	Location	Sample	Factors
CMAI	Cohen-Mansfield et al. (1989) [10]	United States of America (USA)	Nursing home residents, different levels of physical abilities and cognitive decline	1. Aggressive behavior 2. Physically non-aggressive behavior 3. Verbally agitated behavior 4. [Only for day shift: Hiding/hoarding]
	de Jonghe & Kat (1996) [12]	Netherlands	Older patients in psychiatric hospital	1. Physically aggressive behavior 2. Physically non-aggressive behavior 3. Verbally agitated behavior
	Schreiner et al. (2000) [15]	Japan	Nursing home residents in general	4 factors quite similar to the factor structure of Cohen-Mansfield et al (1989) [10], but not clearly labeled
	Choy et al. (2001) [13]	Hong Kong	Inpatients and outpatients with dementia of two hospitals	1. Physically aggressive behavior 2. Physically non-aggressive behavior 3. Verbally agitated behavior
	Suh 2004 [16]	Korea	Nursing home residents with dementia	1. Physically aggressive behavior 2. Physically non-aggressive behavior 3. Verbally agitated behavior 4. Hiding/hoarding
	Rabinowitz et al. 2005 [14]	Australia, New Zealand, USA, Europe, Canada	Nursing home residents with dementia	1. Aggressive behavior 2. Physically non-aggressive behavior 3. Verbally agitated behavior 4. Hiding/hoarding
CMAI-C	Cohen-Mansfield et al. (1995) [17]	United States of America (USA)	Community-dwelling people attending day care centers	1. Verbally non-aggressive behavior 2. Verbally aggressive behavior 3. Physically non-aggressive behavior 4. Physically aggressive behavior
	Weiner et al. (2002) [18]	United States of America (USA)	Community-dwelling people with dementia	No robust factor structure found, i.e. could not replicate the factor structure of Cohen-Mansfield et al (1995) [17]
CMAI-SF	Paudel et al. (2021) [20]	United States of America (USA)	Nursing home residents in general	1. Aggressive behavior 2. Physically non-aggressive behavior 3. Verbally agitated behavior
	Sun et al. (2022) [21]	Taiwan	People with dementia attending day care centers	1. Aggressive behavior 2. Physically non-aggressive behavior 3. Verbally agitated behavior

CMAI: Original 29-item long form of the Cohen-Mansfield Agitation Inventory with a 7-point rating scale of frequency of agitated behaviors [10, 11]; CMAI-C: 36-item Community version of the CMAI with a 7-point rating scale frequency, adapted to the setting of semi-inpatient settings like day care centers [11, 17]; CMAI-SF: 14-item short form of the Cohen-Mansfield Agitation Inventory with a 5-point rating scale of frequency, derived from the original 29-item long form of the CMAI [11, 22]

Recently, an observation tool (CMAI-O) for the original 29-item CMAI was also developed by Griffiths et al. [23] to rate behaviors on a 4-point frequency scale specifically by independent trained observers. The CMAI-O showed adequate internal consistency (α = 61) and convergent as well as discriminant validity with other instruments, while the factor structure was not analyzed.

Cohen-Mansfield et al. also developed a short form (CMAI-SF) based on the factor structure of the CMAI, which includes 14 items, each rated on a 5-point instead of a 7-point frequency scale [11, 22]. It was first described in a study of nursing home residents in which an interrater reliability of 81.8–92.3% was observed [22], and has been used in several studies in recent years [24–30]. Unfortunately, there have been few studies examining construct validity including factor structure. In both nursing home residents in the United States of America (USA) and PlwD attending day care centers in Taiwan, the original three-factor structure consisting of “aggressive behavior”, “physically non-aggressive behavior”, and “verbally agitated behavior” was found (see Table 1).

Because the 14-item CMAI-SF [11, 22] has not been validated in German language, a validation study exploring the construct validity of the measure is scientifically necessary.

Whether the German short form of the instrument can replicate the number and content of the previously published factors remains to be explored. In addition, shared-housing arrangements (SHAs), i.e. innovative, homelike care environments [31], are classified as outpatient settings. However, the conditions are not the same as living in the family structure, but they are also more individual than in nursing homes. Thus, it is unclear whether the three factor structure that has been already found for the CMAI-SF in nursing home [20] and day care [21] can be replicated in the setting of SHAs or whether the four factor structure reported for the original CMAI in semi-inpatient settings such as day-care facilities [17] will be observed.

Therefore, the aim of this study is to explore the construct validity of a German version of the CMAI-SF in people with cognitive impairment in the outpatient setting of German shared-housing arrangements (SHAs).

## Methods

### Design

The data were obtained from the baseline data of the cluster-randomized controlled trial “The DemWG study” [32] to evaluate a non-pharmacological complex intervention for people with dementia and mild cognitive impairment (MCI) in German SHAs (trial registration number: ISRCTN89825211). The sample consisted of *N* = 341 people with dementia or mild cognitive impairment living in 97 SHAs located in nine different German federal states [32]. Residents of the SHAs were included if they had mild-to-moderate dementia according to screening instruments (i.e. Mini Mental State Examination [MMSE] < 24) or MCI (i.e. MMSE > 23, but Montreal Cognitive Assessment [MoCA] < 24). Exclusion criteria were severe dementia (i.e. MMSE < 10), severe auditory or visual impairment, cognitive decline due to diseases other than dementia (e.g. schizophrenia or Korsakoff syndrome), permanent immobility, inability to communicate in the German language, history of more than one stroke, or severe major depression. All study procedures were approved by the Ethics Committee of the University of Bremen (Ref. 2019–18-06–03). Informed consent was obtained before participants were enrolled. A more detailed description of the design and procedures was published by Kratzer et al*.* [32].

### Instruments

Trained nursing staff from the participating SHAs collected data in the form of pseudonymized paper case report forms (CRFs). After completion, the CRFs were sent to the data monitoring committee of the DemWG study and quality checked there. Missing data were requested from the SHAs via mail or phone. Besides the collection of sociodemographic data, the following instruments were administered.

#### Cohen-Mansfield Agitation Inventory-Short Form (CMAI-SF)

The CMAI-SF is a proxy-based instrument for assessing 14 agitated behaviors derived from the original 29-item CMAI [10, 11]. In this validation study, a forward–backward German translation of the CMAI-SF was used because no German translation is currently available. First, a research associate at the University of Bremen translated the English CMAI-SF by Cohen-Mansfield [11, 22] into German. Afterwards, an independent staff member from the Language Center of the Friedrich-Alexander University Erlangen-Nürnberg (FAU) who is a bilingual native speaker translated this version back into English, and a few discrepancies were discussed and corrected in the German version by the research team from the DemWG study. The frequency of each item was rated on a 5-point scale (ranging from 1 to 5), resulting in a total score ranging from 14 to 70 with higher scores indicating more pronounced agitation.

#### Neuropsychiatric Inventory-Nursing Home (NPI-NH)

The German version of the NPI-NH [33, 34] was derived from the Neuropsychiatric Inventory (NPI) by Cummings et al. [35], one of the most widely used instruments for assessing BPSD. It is a proxy-based instrument that is designed to be used by professional caregivers to assess the frequency (5-point scale ranging from 0 to 4) and severity (4-point scale ranging from 0 to 3) of twelve common BPSD. A frequency x severity product score is built for each symptom. The total score is obtained by adding the frequency x severity scores of each item and ranges from 0 to 144 with higher scores indicating more pronounced BPSD.

#### QUALIDEM

The German version 2.0 of the dementia-specific proxy-based Quality of Life (QoL) instrument, the QUALIDEM [36, 37], was administered. It consists of 37 items covering nine dimensions of QoL (subscales), i.e. “care relationship,” “positive affect,” “negative affect,” “restless tense behavior,” “positive self-image,” “social relationships,” “social isolation,” “feeling at home,” and “having something to do.” Every item is rated on a 7-point scale (ranging from “never” to “very frequently”), whereby subscale and total scores are obtained by adding item scores and transforming them into values that range from 0 to 100 [38]. It should be noted that the positively worded items are reverse-scored to line up with the negatively worded items (i.e. “never" corresponds to a score of 0 for a positively worded item and a score of 6 for a negatively worded item). Thus, the higher a subscale score, the higher the quality of life of the PlwD on this dimension.

#### Mini-Mental State Examination (MMSE)

The MMSE [39] is the most widely used cognitive screening test for dementia whose reliability and validity has been established [40–42]. Scores range from 0 to 30, with higher scores indicating higher cognitive functioning.

#### Montreal Cognitive Assessment (MoCA)

The MoCA [43] is a commonly used and widely validated screening tool for MCI [44, 45]. Scores range from 0 to 30, with higher scores indicating higher cognitive functioning.

### Statistical analysis

Data analyses were performed with the “IBM SPSS Statistics 28” software. The complete sample of *N* = 341 participants with baseline data from the DemWG study was available for analysis. Less than 5% had only single missing values from the assessment tools NPI-NH, MMSE, and MoCA, with the exception of the QUALIDEM (*n* = 9, 2.6% with completely missing QUALIDEM scores). Single missing values in the metric scaled data were imputed via iterative random forest imputation [46], whereas for the *n* = 9 (2.6%) with completely missing QUALIDEM scores, only the total QUALIDEM score was imputed via iterative random forest imputation but not any individual item values or subscores. There were 19 cases with a single missing item each (5.6%) in the relevant outcome instrument, the CMAI-SF. These single missing values were also imputed via iterative random forest imputation.

The mean, median, standard deviation, and skewness of participants’ CMAI-SF scores were calculated to describe the distribution of the CMAI-SF score in the current sample.

The factor structure was analyzed in accordance with the latest studies of the CMAI in PlwD [14, 18] and the literature on factor analysis [47]. Requirements for principal component analysis (PCA) were checked with the Kaiser–Meyer–Olkin measure of sampling adequacy, which is recommend to be > 0.60 (Tabachnick & Fidell, 2013) and Bartlett’s test of sphericity for exploring the correlations between items. If the prerequisites were met, a PCA with orthogonal varimax rotation could be conducted. According to the latest studies of the CMAI in PlwD [14, 18], the behavior described in the items had to occur in at least 5% of the sample and items had to have a minimum loading of 0.40 on one of the factors. Only factors with eigenvalues > 1 according to the Kaiser criterion were considered.

An item analysis was conducted, computing the mean, difficulty index, and the discriminatory power of each CMAI-SF item. In addition, the internal consistency of the total CMAI-SF score as well as the identified factors was evaluated by calculating Cronbach’s α.

Construct validity was examined by calculating Pearson correlation coefficients between the CMAI-SF score and the NPI-NH agitation item score (convergent validity) as well as the NPI-NH apathy item score (discriminant validity). We hypothesized that the CMAI-SF would be strongly correlated with the NPI-NH agitation item score, whereas a low correlation with the NPI-NH apathy item score was expected as in previous research [23, 34]. As a sensitivity analysis, we examined whether the CMAI-SF would be positively correlated with the NPI-NH “agitation & restless behavior” factor (items: agitation, disinhibition, irritability, aberrant motor behavior, nighttime behavior disorders) found in the NPI-NH German validation study by Reuther et al. [33]. In order to describe the construct of the CMAI-SF in more detail and to investigate the meaning of agitation with regard to QoL, exploratory correlations between the CMAI-SF score and the QUALIDEM total and subscores were calculated and displayed.

## Results

### Sample

The current sample included 254 PlwD (74.5%) and 87 people with MCI (25.5%), i.e. a total sample of *N* = 341 participants from 97 shared-housing arrangements. The mean age was 82.75 years (*SD* = 8.44), and 76.2% (*n* = 260) were female. Participants’ cognitive impairment severity, as rated by the MMSE and the MoCA, ranged from MCI (*n* = 87, 25.5%, MMSE > 23 & MoCA < 24) to mild (*n* = 111, 32.6%, MMSE 23–18) and moderate dementia (*n* = 113, 33.1%, MMSE 17–10), up to severe dementia (*n* = 30, 8.8%, MMSE < 10). The median time interval between screening and baseline data collection was 3 months (range: 0 to 13 months) due to an interruption in the study because of the outbreak of the COVID-19 pandemic in spring 2020. Therefore, due to the irreversible progression of dementia, people with severe dementia were included in the baseline sample even though severe dementia was a reason for exclusion at screening.

### Distribution of the CMAI-SF score

**Fig. 1:**
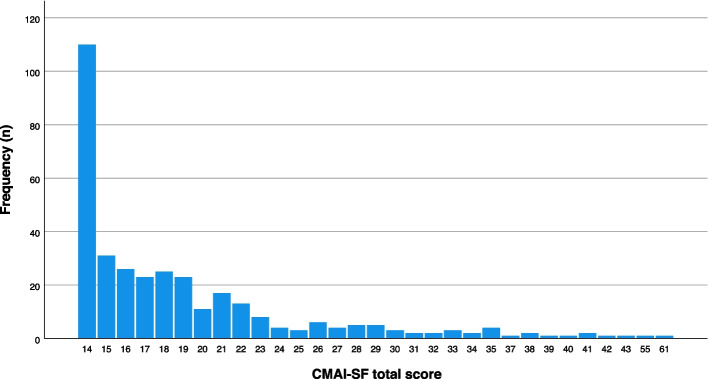
Distribution of the CMAI-SF total score. Illustration of the absolute number of participants (y-axis) with the respective CMAI-SF score (x-axis). Higher scores indicate more pronounced agitation and aggression, range: 14–70

The distribution of the CMAI-SF score covered the range from 14 to 61 points (see Fig. 1). Due to the high frequency of low scores – the 25^th^ percentile was 14 – the distribution was right-skewed (skewness = 2.35). The mean was 18.96 (*SD* = 6.79), and the median was 17. All items appeared in at least 5% of the sample.

### Factor structure

**Fig. 2:**
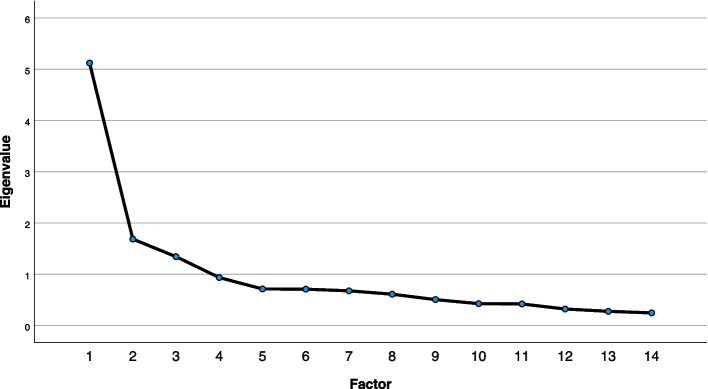
Scree plot for the principal component analysis (PCA) of the CMAI-SF total score

For the PCA, all requirements were fulfilled. The Kaiser–Meyer–Olkin measure of sampling adequacy was 0.832, and Bartlett’s test of sphericity was significant (*p* < 0.001), indicating that correlations between items were large enough to perform a PCA. Examinations of the Kaiser criterion and the scree plot (Fig. 2) provided empirical justification for retaining 3 factors with eigenvalues exceeding 1, accounting for 58.2% of the total variance in the CMAI-SF score. Table 2 presents the loadings on the three factors.

**Table 2:** Factor loadings for a PCA with varimax and promax rotations

	**Varimax rotation**			**Promax rotation**		
**Variables**	**Factor 1**	**Factor 2**	**Factor 3**	**Factor 1**	**Factor 2**	**Factor 3**
Item 4: other aggressive behaviors or self-abuse including: intentional falling, making verbal or physical sexual advances, eating/drinking/chewing inappropriate substances, hurt self or other	.818			.886		
Item 2: hitting, kicking, pushing, biting, scratching, aggressive spitting	.783			.805		
Item 3: grabbing onto people, throwing things, tearing things, or destroying property	.706			.696		
Item 12: strange noises (weird laughter or crying)	.695			.713		
Item 1: cursing/verbal aggression	.612	.427		.630		
Item 9: constant request for attention or help		.787			.875	
Item 10: repetitive sentences, calls, questions, or words		.783			.855	
Item 11: complaining, negativism, refusal to follow directions	.414	.629			.618	
Item 6: general restlessness, performing repetitious mannerisms, tapping, strange movements		.510	.474		.473	
Item 14: screaming		.484			.469	
Item 7: inappropriate dress or disrobing			.816			.835
Item 13: hiding things, hoarding things			.672			.711
Item 8: handling things inappropriately			.668			.640
Item 5: pace, aimless wandering, trying to get to a different place (e.g. out of the room/building)		.436	.635			.584

Includes only factor loadings of .40 or above

Because the PCA with a varimax rotation produced ambiguous factor loadings for Items 1, 11, 6, and 5, a promax rotation was used in accordance with Rabinowitz et al. [14]. The promax rotation resulted in an unambiguous factor structure (see Table 2).

Factor 1 consisted of Items 1, 2, 3, 4, and 12 (i.e. aggressive behavior; *verbal and physical*). Factor 2 consisted of Items 6, 9, 10, 11, and 14 (i.e. verbally agitated behavior + Item 6: general restlessness, repetitious mannerisms, tapping, strange movements), and Factor 3 consisted of Items 5, 7, 8, and 13 (i.e. physically non-aggressive behavior).

### Item analysis

The mean item difficulty of the CMAI-SF was 0.09 and ranged from 0.02 to 0.18 (see Table 3). Along with the mean item values, which ranged from 1.07 to 1.70 (item range: 1 to 4), the difficulty suggested that agitation was low at the item level (see Table 3). All items showed moderate to high discriminatory power [48] ranging from 0.37 to 0.62 regarding the total CMAI-SF (see Table 3). Specific to each factor, the items also showed moderate to high [48] discriminatory power ranging from 0.43 to 0.71 (see Table 3).

**Table 3:** Characteristics of the CMAI-SF items

Item summary	Mean (SD)	Item difficulty	Discrim-inatory power (Total Score)^a^	Discrim-inatory power (Factor)^b^	Cronbach’s alpha “if item deleted” (Total score)^a^	Cronbach’s alpha “if item deleted” (Factor)^b^
**Factor 1 (aggressive behavior)**						
Item 4: other aggressive behaviours or self abuse including: intentional falling, making verbal or physical sexual advances, eating/ drinking/ chewing inappropriate substances, hurt self or other	1.07 (0.32)	.02	.43	.62	.84	.71
Item 2: hitting, kicking, pushing, biting, scratching, aggressive spitting	1.13 (0.50)	.04	.54	.71	.84	64
Item 3: grabbing onto people, throwing things, tearing things, or destroying property	1.16 (0.58)	.04	.54	.60	.83	.67
Item 12: strange noises (weird laughter or crying)	1.09 (0.42)	.02	.47	.50	.84	.72
Item 1: cursing/verbal aggression	1.64 (1.00)	.17	.46	.50	.84	.80
**Factor 2 (verbally agitated behavior)**						
Item 9: constant request for attention or help	1.70 (1.16)	.18	.50	.57	.84	.70
Item 10: repetitive sentences, calls, questions, or words	1.52 (1.09)	.14	.49	.60	.84	.69
Item 11: complaining, negativism, refusal to follow directions	1.54 (0.96)	.14	.62	.56	.83	.70
Item 6: general restlessness, performing repetitious mannerisms, tapping, strange movements	1.34 (0.90)	.09	.61	.51	.83	.72
Item 14: screaming	1.18 (0.64)	.05	.48	.44	.84	.75
**Factor 3 (physically non-aggressive behavior)**						
Item 7: inappropriate dress or disrobing	1.34 (0.88)	.09	.53	.66	.83	.59
Item 13: hiding things, hoarding things	1.45 (0.93)	.12	.37	.43	.84	.72
Item 8: handling things inappropriately	1.31 (0.75)	.08	.55	.54	.83	.67
Item 5: pace, aimless wandering, trying to get to a different place (e.g. out of the room/building)	1.49 (1.08)	.13	.55	.50	.83	.70

^a^ regarding the CMAI-SF total score; ^b^ regarding the relevant subscore of the CMAI-SF factor

### Internal consistency

The entire CMAI-SF had a high [49] Cronbach’s α of 0.85, and for each of the 14 items, Cronbach’s α “if item deleted” was below α for the total score. Specific to each factor, Cronbach’s α was acceptable for Factor 1 (α = 0.75), Factor 2 (α = 0.76), and Factor 3 (α = 0.73), and Cronbach’s α “if item deleted” for each item was always below the factor-specific α (see Table 3).

### Construct validity

As suspected, the CMAI-SF score was strongly correlated with the NPI-NH agitation item (*r* = 0.66, *p* < 0.001) and the NPI-NH factor “agitation & restless behavior” (*r* = 0.82, *p* < 0.001). Thus, convergent validity was confirmed. The correlation between the CMAI-SF and the NPI-NH apathy item was low (*r* = 0.28, *p* < 0.001) but still significant. Therefore, discriminant validity was confirmed.

Beyond these findings, QoL decreased significantly with agitation, as there was a moderate negative correlation between the CMAI-SF and QUALIDEM total scores (*r* = -0.35, *p* < 0.001).

There were also low to moderate negative correlations between the CMAI-SF and the QUALIDEM subscores “positive affect” (-0.36, *p* < 0.001), “negative affect” (*r* = -0.33, *p* < 0.001), “positive self-image” (*r* = -0.19, *p* < 0.001), “social relationships” (*r* = -0.31, *p* < 0.001), “social isolation” (*r* = -0.44, *p* < 0.001), “feeling at home” (*r* = -0.39, *p* < 0.001), and “having something to do” (*r* = -0.27, *p* < 0.001). High correlations were found between the CMAI-SF and the QUALIDEM subscores “care relationship” (*r* = -0.51, *p* < 0.001) and “restless tense behavior” (*r* = -0.59, *p* < 0.001).

## Discussion

In this first validation of the German version of the CMAI-SF, an unambiguous three-factor structure with acceptable internal consistency was confirmed with a PCA. Almost all item loadings were above 0.50, with the exception of two items loading above 0.46. The internal consistency of the total score form was high.

The three factor structure (“aggressive behavior”, “physically non-aggressive behavior”, and “verbally agitated behavior”) that has been already found for the CMAI-SF in nursing home [20] and day care samples [21] could be replicated in SHAs using the German version of the CMAI-SF. The first factor was largely consistent with the factor "aggressive behavior" found in the validation of the original 29-item CMAI by Cohen-Mansfield et al. [10] and Rabinowitz et al. [14] in nursing home samples, except that in these two previous studies, the item "strange noises" did not load on any factor, and in Cohen-Mansfield et al. [10], the item “other aggressive behaviors” could not be assigned to one factor because these behaviors appeared in less than 5% of the sample. The second factor in our study was largely consistent with the “verbally agitated behavior” factor from the original 29-item CMAI found by Cohen-Mansfield et al. [10] and Rabinowitz et al. [14], with the exception that in the present work, Item 6 "general restlessness, performing repetitive mannerisms, tapping, strange movements" also loaded on this factor. The content of this item includes behaviors that reflect physical agitation. However, the factor loading of this item constituted one of the two lowest loadings from the questionnaire (< 0.50); when we applied a varimax rotation, the ambiguous item had similar loadings on Factor 2 and the content-matching Factor 3. To avoid this ambiguity, we decided to use a promax rotation in the final version. The third factor was also largely consistent with the “physically non-aggressive behavior” factor found in the validation of the original 29-item CMAI by Cohen-Mansfield et al. [10] and Rabinowitz et al. [14] except that Item 6 "general restlessness, performing repetitive mannerisms, tapping, strange movements" did not load on this factor when a promax rotation was used. In our study, Item 13 “hiding things, hoarding things” also loaded on Factor 3 “physically non-aggressive behavior” just as it had in the day-care sample [17] and did not emerge as a fourth factor as it had in some nursing home validation samples [14–16]. In summary, with a few exceptions, the factor structure closely resembled the structure found by Cohen-Mansfield et al. [10] and Rabinowitz et al. [14] in their nursing home samples using the original CMAI.

However, the assignment of Item 6 remained unsettled because the ambiguous results from the varimax rotation also showed that Item 6 loaded on the content-matching Factor 3 ("physically non-aggressive behavior"), but the results from the promax rotation indicated that Item 6 loaded on Factor 2 (“verbally agitated behavior”). Therefore, future research should further investigate the results of this exploratory factor analysis. In fact, Sun et al. [21] in their validation of a Chinese version of the CMAI-SF also found that item 6 loaded on the "verbally agitated behavior" factor – they explained this by considering that people living with dementia express their demands or unmet needs in part through agitation, tapping, and strange movements, which could therefore be classified as "verbally aggressive."

The four-factor structure found in day-care centers by Cohen-Mansfield et al. [17] did not appear in the present work, which is plausible considering that people in SHAs (i.e. our sample) live in the care environment 24/7, thus closely resembling life in a nursing home. Indeed, SHAs are seen as an alternative to a nursing home, when living at home is no longer possible [50]. In addition, even if a high degree of autonomy and self-determination is guaranteed to the residents in SHAs, the residents live in a care environment with professional care and not at home [31, 50, 51]. This resemblance to a nursing home is further supported by the fact that we detected a factor structure in our sample, whereas it was not possible for Weiner et al. [18] to detect a factor structure for community-dwelling PlwD because several of the CMAI items had a frequency below 5% in this subgroup. Nonetheless, using the CMAI-SF, Sun et al. [21] also found the original three-factor structure in people with dementia in day care settings, so it could also be concluded that the CMAI-SF in particular has a three-factor structure both in the nursing home and in semi-inpatient settings such as day care. Further research is needed to sufficiently clarify this issue.

Internal consistency, measured with Cronbach’s α, was good for the total CMAI-SF and acceptable for the three factors. All items increased the internal consistencies of the CMAI-SF total score and the three factor scores: For all items, Cronbach’s α “if item deleted” was below Cronbach’s α for the complete scale or the factors. All the CMAI-SF items showed moderate to high discriminatory power, whereas item difficulty was rather low. These results suggest that people with MCI and mild to moderate dementia in German SHAs had relatively low agitation levels. However, all 14 items were relevant in the sense that each behavior was described in at least 5% of the PlwD by the proxy raters.

With respect to construct validity, the convergent validity of the CMAI-SF was confirmed. As hypothesized, there were significant and high correlations between the CMAI-SF total score and the NPI-NH agitation item as well as the NPI-NH “agitation & restless behavior” factor [33]. Furthermore, QoL decreased significantly as agitation/aggression increased, as there were low to moderate significant negative correlations between the CMAI-SF total score and QUALIDEM total and subscores. The discriminant validity of the CMAI-SF was confirmed, as there was a low but significant positive correlation between the CMAI-SF total score and the NPI-NH apathy item (as hypothesized on the basis of Griffiths et al*.*, 2020) [23].

### Limitations

The present study was the first study to validate the German version of the CMAI-SF in a large sample of clusters (SHAs) and participants. The sample also covered the broad spectrum of cognitive impairments ranging from MCI to severe dementia. In addition, the use of the validated German version of the NPI-NH enabled us to investigate convergent validity with the NPI-NH agitation item and the “agitation & restless behavior” factor from the NPI-NH. Nevertheless, the present study has some limitations. First, the present validation was based on data that were actually collected for an RCT and not for the purpose of validating the CMAI-SF. Furthermore, the present analysis is only an exploratory factor analysis, so the results should be considered hypothesis-generating only, and further research using confirmatory factory analysis is needed to conclusively investigate the factor structure of the CMAI-SF. In addition, the results cannot be readily generalized to other settings, so further studies are needed to test the factor structure of the CMAI-SF in other settings as well. Last but not least, a future study should be conducted in which both the original 29-item CMAI and the 14-item CMAI-SF are examined for concurrent validity in the same sample.

## Conclusion

The present study is the first study to validate a German version of the CMAI-SF, the 14-item short form of the CMAI. The results show that the CMAI-SF is a time-efficient, reliable, and valid scale for assessing agitation in people with MCI and dementia in SHAs. In our exploratory factor analysis, we identified three factors, which were similar to the factors “aggressive behavior,” “verbally agitated behavior,” and “physically non-aggressive behavior” already found in nursing home samples for the original CMAI and the CMAI-SF and in day care samples for the CMAI-SF. Thus, the findings provide preliminary evidence that the CMAI-SF can be used instead of the long-form CMAI to reduce time, costs, and the burden on proxy raters, when investigating agitation and aggression in people with dementia and MCI in future trials. However, further research is needed to confirm these results.

## Data Availability

The datasets generated during and/or analysed during the current study will be available upon request from Stephan Kloep (kloep@uni-bremen.de). Data will be available in the time interval from 12 months until 36 months after publication of the article. The data will be provided for non-commercial research purposes only to researchers with a proposal that was peer-reviewed and approved by an independent review committee. The inquiring researchers have to present an analysis plan and state the research purpose for which the data are needed, e.g. meta-analysis. Data will be available through the data warehouse of the University Bremen without any additional investigator support. The data that can be provided refer solely to the data underlying the presented results of the manuscript. They will be completely anonymized, linkage to the stored data with personal information will not be possible, thus case-specific additional information/clarification cannot be provided anymore.

## Abbreviations

BPSD: Behavioral and psychological symptoms of dementia
CMAI: Cohen-Mansfield Agitation Inventory
CMAI-SF: Cohen-Mansfield Agitation Inventory-Short Form
FAU: Friedrich-Alexander-Universität Erlangen-Nürnberg
MCI: Mild cognitive impairment
MMSE: Mini-Mental State Examination
MoCA: Montreal Cognitive Assessment
NPI-NH: Neuropsychiatric Inventory – Nursing Home Version
PCA: Principal component analysis
QoL: Quality of Life
PlwD: People living with Dementia
RCT: Randomized controlled trial
SHA: Shared-housing arrangement
